# The role of ADAM17 in cerebrovascular and cognitive function in the APP/PS1 mouse model of Alzheimer’s disease

**DOI:** 10.3389/fnmol.2023.1125932

**Published:** 2023-03-02

**Authors:** Yanna Tian, Katie Anne Fopiano, Vadym Buncha, Liwei Lang, Hayden A. Suggs, Rongrong Wang, R. Daniel Rudic, Jessica A. Filosa, Zsolt Bagi

**Affiliations:** ^1^Department of Physiology, Medical College of Georgia, Augusta University, Augusta, GA, United States; ^2^Department of Pharmacology, Medical College of Georgia, Augusta University, Augusta, GA, United States

**Keywords:** a disintegrin and metalloproteinase 17, Alzheimer’s disease, vasodilation, cognitive decline, mouse model

## Abstract

**Introduction:**

The disintegrin and metalloproteinase 17 (ADAM17) exhibits α-secretase activity, whereby it can prevent the production of neurotoxic amyloid precursor protein-α (APP). ADAM17 is abundantly expressed in vascular endothelial cells and may act to regulate vascular homeostatic responses, including vasomotor function, vascular wall morphology, and formation of new blood vessels. The role of vascular ADAM17 in neurodegenerative diseases remains poorly understood. Here, we hypothesized that cerebrovascular ADAM17 plays a role in the pathogenesis of Alzheimer’s disease (AD).

**Methods and results:**

We found that 9-10 months old APP/PS1 mice with b-amyloid accumulation and short-term memory and cognitive deficits display a markedly reduced expression of ADAM17 in cerebral microvessels. Systemic delivery and adeno-associated virus (AAV)-mediated re-expression of ADAM17 in APP/PS1 mice improved cognitive functioning, without affecting b-amyloid plaque density. In isolated and pressurized cerebral arteries of APP/PS1 mice the endothelium-dependent dilation to acetylcholine was significantly reduced, whereas the vascular smooth muscle-dependent dilation to the nitric oxide donor, sodium nitroprusside was maintained when compared to WT mice. The impaired endothelium-dependent vasodilation of cerebral arteries in APP/PS1 mice was restored to normal level by ADAM17 re-expression. The cerebral artery biomechanical properties (wall stress and elasticity) and microvascular network density was not affected by ADAM17 re-expression in the APP/PS1 mice. Additionally, proteomic analysis identified several differentially expressed molecules involved in AD neurodegeneration and neuronal repair mechanisms that were reversed by ADAM17 re-expression.

**Discussion:**

Thus, we propose that a reduced ADAM17 expression in cerebral microvessels impairs vasodilator function, which may contribute to the development of cognitive dysfunction in APP/PS1 mice, and that ADAM17 can potentially be targeted for therapeutic intervention in AD.

## 1. Introduction

Cardiovascular disease is a common comorbidity in Alzheimer’s disease (AD; Gorelick et al., 2011; Iadecola, 2013; Corriveau et al., 2016). Notably, 50% of clinically diagnosed AD patients display a mixed vascular and AD pathology (Snowdon et al., 1997; Santisteban and Iadecola, 2018). Cardio-and cerebrovascular diseases are multifactorial conditions, increasingly prevalent in older adults with hypertension and type 2 diabetes that are risk factors for dementia (Udelson, 2011; Goyal et al., 2016; Silverman et al., 2016). It is now believed that cerebrovascular changes not only accompany but are also mechanistically linked to the development of AD. In support, human autopsy findings demonstrate that a significant portion of clinically diagnosed AD patients have histopathology-defined microvascular brain injury (Bagi et al., 2018; Park et al., 2018). Consistent with the contribution of cerebral microvascular dysfunction to human AD, we recently described selective vasodilator dysfunction of cerebral parenchymal arterioles, which appears to be associated with AD neuropathological changes and MRI-defined cerebral microstructural changes in brain donors with no pathologically described macroscopic infarcts or hemorrhages (Bagi et al., 2022). Presently unresolved is the extent to which cerebrovascular dysfunction is associated with memory deficits and cognitive decline in AD patients. Furthermore, the molecular underpinnings through which cerebrovascular dysfunction contributes to the development of the AD pathomechanism is incompletely understood, and therefore preventative therapeutic measures cannot be adapted.

A disintegrin and metalloproteinase 17, ADAM17 (also known as tumor necrosis factor (TNF)-α converting enzyme or TACE), was initially described as a sheddase for cell membrane-bound TNF (Gooz, 2010). ADAM17 was later identified as a highly promiscuous enzyme regulating multiple substrates by proteolytic cleavage, such as TNF-α receptors, pro-inflammatory and inflammation resolution mediators, as well as vascular cell membrane-bound adhesion molecules, growth and angiogenic factors (Gooz et al., 2009). Interestingly, a prior report has shown that a rare genetic variant, leading to a loss-of-function in ADAM17, is associated with the pathogenesis of AD in humans (Hartl et al., 2020). It is known that pathological amyloidogenic amyloid beta (Aβ) accumulation, due to abnormal processing of the amyloid precursor protein (APP), promotes AD development *via* neurotoxic effects (Allinson et al., 2003; Sastre et al., 2008), and also, in part, by causing cerebrovascular impairments (Sastre et al., 2008; Iadecola, 2017). ADAM17, owing to its α-secretase activity, has been implicated in the shedding of the APP, promoting a soluble, non-amyloidogenic fragment formation (Pietri et al., 2013). However, the role of ADAM17 in the development of cerebrovascular and cognitive impairments in AD remains incompletely understood. In this study, we set out to examine the role of ADAM17 in contributing to vascular and cognitive impairments by using an established mouse model of AD, the APP/PS1 mice.

## 2. Methods

### 2.1. Animals

The work involving experimental animals was conducted under the protocol approved by the Institutional Animal Care and Use Committee at the Medical College of Georgia, Augusta University. All experimental animal procedures performed in this study were in compliance with the European Convention for the Protection of Vertebrate Animals used for Experimental and other Scientific Purposes. Experiments were carried out in nine to 10-month-old male and female APP/PS1 mice, which are double transgenic mice expressing a chimeric mouse/human amyloid precursor protein (Mo/HuAPP695swe) and a mutant human presenilin 1 (PS1-dE9), both directed to CNS neurons (MMRRC strain: 034832-JAX, The Jackson Laboratory; Jankowsky et al., 2004). The mice are on the C57BL/6J genetic background. The mice were housed in the animal care facility and accessed rodent chow and tap water *ad libitum* with a 12-h light:dark cycle.

### 2.2. Animal behavioral tests

Animal behavioral tests were conducted in the small animal behavior core lab in Augusta University.

#### 2.2.1. Morris water maze

The Morris Water Maze was used to test spatial learning and memory (Vorhees and Williams, 2006; Bromley-Brits et al., 2011). The water-maze apparatus consisted of a circular pool of 150 cm in diameter and 60 cm in height, filled with water made opaque with non-toxic white paint, kept at a temperature of 22°C. Direct light to the pool was avoided. Different signs were placed around the pool to help the mice to memorize direction. Individual mice received a five-day acquisition training, consisting of four trials per day. In each training trial, a plastic transparent platform was placed 1.5 cm below the water surface; the mice were placed into the pool facing the wall and were allowed to explore the pool to look for the platform until finding the platform, for 90 s. If the mice failed to find the platform within 90 s, they were guided to the platform. The mice were allowed to stay on the platform for 20 s. If the mouse failed to swim for the trial duration, they were removed and allowed a second trial. If upon the second trial, the mouse again failed to swim, it was excluded from the data set results. Velocity of mice swim times were analyzed to assess differences in movement and locomotion. On each trial, the mouse started from a different location across trials and days. After 5 days of acquisition training sessions, the platform was removed from the pool and each mouse was tested in a single 60 s probe trial. Two days after the probe trial, to exclude the mice with vision problems, each mouse was tested in 3 trials with a visible red flag on the platform. The mice which were not be able to find the platform within 60 s were excluded. The swimming path of the mice was recorded by a computer-based video-tracking system and the time spent to find the platform and the target quadrant were recorded.

#### 2.2.2. Spontaneous Y-maze

The spontaneous Y-maze test is used to measure spatial working memory (Kraeuter et al., 2019). The maze consists of three arms (41 cm long, 16 cm high, and 9 cm wide, labeled A, B, or C) diverging at a 120̊ angle from the central point. The experiments were performed in a dimly illuminated room and the floor of the maze was cleaned with hypochlorous water-soaked paper after each mouse to avoid olfactory cues. Each mouse was placed at the end of the starting arm and allowed to move freely through the maze during a 5-min session. The sequence of arm entries was recorded and the number of arm entries was manually counted in a blinded manner; a mouse was considered to have entered an arm when all four paws were positioned in the arm runway. An actual alternation was defined as entries into all of the three arms on consecutive occasions. The maximum alternation was subsequently calculated by measuring the total number of arm entries minus 2 and the percentage of alternation was calculated as (actual alternation/maximum alternation)*100. Total number of arms entered during the sessions (reflecting locomotor activity) was also recorded. Mice that entered arms less than eight times during the test were eliminated because the data obtained from those mice were not considered to be representative.

#### 2.2.3. Novel objective recognition

The Novel Objective Recognition (NOR) test was performed similarly as described before (Lueptow, 2017). Mouse behavior is recorded with a video camera. On each day before the experiment, the mice were brought to the testing room 30 mins before starting testing to pre-habituate with the environment. One day preceding the test, after 30 mins habituation, each mouse was allowed to freely explore the box with no objects for 8 mins. On the testing day, two identical objects were placed at two opposite positions within the box at the same distance from the nearest corner. Each mouse was allowed to explore the identical objects for 10 mins, and then returned to their home cage for 4 h. Then, one familiar object and one novel object were placed in the box. Four hours later, each mouse was allowed to explore the objects for 10 mins. Novel objects were identical in size but different in shape and color to the familiar objects. All objects were fixed on the bottom of the box to avoid movement. Box and objects were cleaned with hypochlorous water-soaked paper between each animal. The time mice spent exploring each object was recorded using two stop watches and measured manually. Animals with a total exploration time of less than 3 s during the testing session were excluded from analysis. The location preference in the training phase and recognition index (RI) for (N/F) in the testing phase were calculated using the following formula: Recognition index (RI; N/F) = time exploring novel object / (time exploring novel object + time exploring familiar object) × 100%. Delta Novel – Familiar = (time exploring the novel object – time exploring the familiar objects) / (time exploring novel object + time exploring familiar object) × 100%.

### 2.3. Adeno-associated virus injection

Recombinant adeno-associated virus 9 (AAV9) was constructed and purchased from VectorBuilder. Mouse ADAM17-mCherry-AAV9 and eGFP-AAV9 were used in the following experiments. Mouse ADAM17-mCherry-AAV9, or eGFP-AAV9 controls, were used to overexpress ADAM17 in the APP/PS1 mice through intravenous tail-vein injections (particle number: 1 × 10^11^ of AAV9 diluted in 200 μL sterile PBS, total volume of 300 μL injected). Mice were assessed for functional and morphological endpoints 3 months after injections.

### 2.4. Purification of brain microvessels

The method used for mice brain microvessel isolation was performed as described previously (Paraiso et al., 2020). Briefly, the brain tissue was placed in a glass tube containing 8 mL of ice-cold HBSS solution (14025-076, Gibco) and homogenized. The brain homogenate was mixed with an equal volume of 40% Ficoll (17030010, Cytiva) to a final concentration of 20% Ficoll in HBSS solution. The tubes were shaken before centrifugation and the resulting homogenate was centrifuged at 5,800g for 20 mins at 4°C. The pellet was gently washed with 5 mL of 1% BSA/HBSS (BP1600, Fisher BioReagents). It was then filtered with 300 μm nylon mesh and the flow through was washed with 1% BSA/HBSS. The mixture was filtered through a 30 μm strainer, with a speed of 1 drop every 2 s. The brain microvessels were captured on top of the strainer. The 30 μm strainer was inverted and rinsed with 10 mL of 1% BSA/HBSS, to wash the vessels down. The wash-through was centrifuged at 5,800g for 20 mins at 4°C. The supernatant was discarded and the vessels pellet was saved for further analysis.

### 2.5. Western blot

Whole brain samples and the isolated brain microvasculature were homogenized in radio-immunoprecipitation assay (RIPA, R0278, Sigma) buffer mixed with 1% protease inhibitor cocktail (P8340, Sigma). Protein concentration was measured by Bradford assay. Equal amounts of protein were loaded for gel electrophoresis. After blotting, membranes (Hybond-P, GE Healthcare) were probed with a rabbit polyclonal anti-ADAM17 antibody (1:1000, ab2051, Abcam), followed by incubation with a HRP linked secondary antibody (anti-rabbit IgG, 7074S, Cell Signaling). Enhanced chemiluminescence was visualized autoradiographically by ChemiDoc XRS+ (170-5061, BioRad). Protein expression was normalized to β-actin (1:1000, 8H10D10, Cell Signaling).

### 2.6. Videomicroscopic assessment of isolated cerebral arteries of the mouse

Mice, under deep anesthesia, were decapitated and the brain was removed. The bottom section of the brain was placed in ice-cold artificial cerebral spinal fluid (ACSF) solution, equilibrated with a gaseous mixture of 10% O_2_-5% CO_2_ – balanced nitrogen, at a pH of 7.4. Mice were euthanized by exsanguination and removal of the aorta; other vital organs were saved and used for further assessments. With the use of microsurgical instruments and an operating microscope, the posterior cerebral artery was isolated and transferred into an organ chamber containing two glass micropipettes, filled with ACSF solution. The artery was cannulated at both ends and the micropipettes were connected with silicone tubing to a hydrostatic reservoir to set the intraluminal pressure to 50 mmHg. Artery preparations were incubated for a 1-h period; spontaneous arterial tone developed in response to 50 mmHg pressure during the incubation period, if spontaneous tone did not develop during the incubation period, U46619 (~10^-8^ M) was applied to the arteries to induce an approximately 25% tone. Arteries that did not develop a spontaneous tone or could not be preconstricted were excluded from the analyses. After the incubation period, diameter changes were measured with a videocaliper (Colorado; Bagi and Koller, 2003; Erdei et al., 2006) in response to cumulative concentrations of the endothelium-dependent agonist, acetylcholine (ACh, 10^-9^–10^-6^ M, Sigma), and to the direct vascular smooth muscle acting, nitric oxide (NO) donor, sodium nitroprusside (SNP, 10^-9^–10^-5^ M, Sigma).

At the end of the experiments, the internal and outer artery diameter was measured in response to incremental increases in intraluminal pressure from 10 to 70 mmHg, in 20 mmHg increments, in the absence of extracellular calcium (calcium-free ACSF solution). The normalized artery diameter was calculated (as measured at 10 mmHg intraluminal pressure). The wall stress (s) was calculated as follows: s = P × D/2WT, where P is pressure and 1 mmHg = 1334 dyne/cm^2^, and as described (Ohanian et al., 2014). The incremental elastic modulus (Einc) was calculated as follows: Einc = (Δp/Δro)2rix2ro/(ro2−ri2), where ri is the inner, ro is the outer radius, Δro is the change in outer radius in response to intraluminal pressure change of Δp, as described (Mericli et al., 2004).

### 2.7. Immunohistochemistry

Mice were anesthetized using isoflurane (1-3%) and were perfused transcardially with 20 mL of room temperature 0.01 M PBS followed by 20 mL of cold 4% formaldehyde (sc-281692, Santa Cruz Biotechnology). Then, the mouse brains were dissected and post-fixed in 4% formaldehyde overnight at 4°C. Brains were cryoprotected with 30% sucrose in 0.01 M PBS for a minimum of 72 h. The brains were cut into 40 μm thick cryosections and saved in a cryoprotectant solution (50 mM PBS, ethylene glycol, and glycerol) at −20°C. For immunohistochemical staining, brain slices located at +1.32, −0.82 (cortex), and −1.64 −2.92 (hippocampus and cortex) were used. After being removed from the cryoprotectant solution, brain slices were washed with 0.01 M PBS three times at room temperature, 45 mins per wash. Brain slices were permeabilized with 0.01 M PBS containing 0.5% Triton X-100 (T8532, Sigma) for 10 mins at room temperature. Brain slices were blocked with 0.01 M PBS containing 10% normal horse serum for 1 h at room temperature. Primary antibodies were applied in 0.01 M PBS containing 0.3% Triton X-100 for 48 h at 4°C. To identify β-amyloid plaques, the primary chicken anti-amyloid beta antibody was used (1:1000, AP31802PU-N, Origene) followed by incubation with secondary antibody (1: 3000, anti-chicken, AlexaFluor^®^555) for 4 h at room temperature. DAPI (H-2000, Vector Laboratories) was used for nuclear staining. Structured illumination microscopy (SIM-Apotome, AxioImagerM2, CarlZeiss) was used for immunofluorescent detection. The analysis of positive β-amyloid staining was quantified using ImageJ.

### 2.8. Automatic 3-dimensional vessel network reconstruction and quantification

For cerebral vascular network reconstruction, the thickly cut brain slice sections (40 μm) described in the previous section were immuno-labeled with Tomato-Lectin DyLight 594 Antibody (Vector Laboratories, DL-1177, 1:150, overnight, 4°C). DAPI was used for nuclear staining. Structured illumination microscopy (SIM-Apotome, AxioImagerM2, CarlZeiss) was used for immunofluorescent detection. Images were taken using z-stack imaging, with images taken every 0.5 μm, using 20X magnification for vascular reconstruction. Per animal, three fields of view were taken using z-stack imaging and averaged. After obtaining z-stack images *via* immunofluorescence microscopy, image stacks were uploaded into Vesselucida360^®^ software for unbiased reconstruction and consequent analysis. Vessels were reconstructed using the Automatic Rayburst Crawl tracing option with vessel tracing set at a sensitivity of 70. Refinement of tracing seeds was between 1 and 3 based on the sample. User-guided manual tracing was used following automatic tracing, with the Rayburst Crawl tracing option, followed by manual tracing of any remaining vessels. Reconstructions were exported to the Vesselucida Explorer^®^ software for automatic quantification of vessel network parameters.

### 2.9. Liquid chromatography and mass spectrometry

For Mass Spectrometry, whole brain samples were homogenized in radio-immunoprecipitation assay (RIPA, Sigma) buffer mixed with 1% protease inhibitor cocktail (Sigma). Afterwards, 50 μg of protein per sample was sent to the Proteomics Core Laboratory at the Medical College of Georgia for protein analysis *via* liquid chromatography mass spectrometry analysis (LC-MS/MS). Samples were digested for 16 h with trypsin at 37°C, trifluoroacetic acid was added to a final concentration of 0.1% (v/v) to stop the digestion. Samples were centrifuged at 15000g for 5 mins and supernatants were used for the LC-MS analysis. Digested peptide samples were analyzed on an Orbitrap Fusion tribrid mass spectrometer (Thermo Scientific), with an Ultimate 3000 nano-UPLC system (Thermo Scientific) connected. Peptide samples were first trapped on a Pepmap100 C18 peptide trap (5 μm, 0.3 × 5 mm). Cleaned peptides were further separated on a Pepman 100 RSLC C18 column (2.0 μm, 75 μm × 150 mm) at 40°C, using a gradient of 2% to 40% acetonitrile with 0.1% formic acid over 120 min at a flow rate of 300 nL/min. Eluted peptides were introduced into the Orbitrap Fusion MS *via* nano-electrospray ionization source at a temperature of 300 °C and a spray voltage of 2000 V. The peptides were then analyzed in the MS using data-dependent acquisition in positive mode with the Orbitrap MS analyzer for precursor scans at 120,000 FWHM from 400 to 1600 m/z (with quad isolation) and the ion-trap MS analyzer for MS/MS scans at top-speed mode (3-s cycle time), with dynamic exclusion settings (repeat count 1 and exclusion duration 15 s). Higher-energy C-trap dissociation (HCD) was used to fragment the precursor peptides with a normalized energy level of 30%. Raw MS data were processed *via* the Proteome Discoverer software (ver 1.4) and submitted for SequestHT algorithm against the SwissProt mouse protein database. SequestHT search parameters were set as 10 ppm precursor and 0.6 Da product ion mass tolerance, with static carbidomethylation (+57.021 Da) for cysteine and dynamic oxidation for methionine (+15.995 Da). The percolator peptide spectrum matching (PSM) validator algorithm was used for PSM validation. Proteins unable to be distinguished based on the database search results were grouped to satisfy the principles of parsimony. Proteomic data was analyzed for further analysis if the ΣPSM value ≥2 per group analyzed (WT, eGFP, ADAM17) using unpaired student t-test between two group comparisons. Principal component analysis was run using AtlAnalyze software. Statistically significant (*p* < 0.05) proteins identified were used in heat-map analysis (-log_10_(*p*-value) vs WT) and for further pathway analysis. Gene Ontology pathway analysis with statistically significant proteins was performed (Panther Classification System). For GO pathway analysis the following parameters were used: Analysis Type: PANTHER Overrepresentation Test; Annotation Version: GO Ontology database; Reference List: Mus musculus (all genes in database); Annotation Data Set Used: GO Biological Process complete, GO Molecular Function complete, GO Cellular Component complete; Test Type: Fisher’s Exact; Correction: Calculated False Discovery Rate (FDR). GO pathways identified with an FDR < 0.05 were used for further analysis. GO pathway analysis was visualized in scatterplot format using multidimensional scaling (MSD) whereby semantically similar GO pathways can be visualized in close proximity (Revigo).

### 2.10. Statistics

All statistical analyses were performed using GraphPad Prism Software. Data were drawn to analyze after being tested for normality using Kolmogorov-Smirnov test. When meeting normality data comparisons between groups were analyzed by two-way repeated-measures ANOVA followed by Sidak’s multiple comparisons test, or with a two-tailed, unpaired Student t-test, as appropriate. A non-parametric Kruskal-Wallis test was used when normality assumptions were not met. Data are expressed either as mean±SEM or box-and-whisker plots, in which the minimum, the 25th percentile, the median, the 75th percentile, and the maximum are presented, and + indicates the mean of values. *P* < 0.05 was considered statistically significant.

## 3. Results

### 3.1. APP/PS1 mice display impairment in short term memory

**Figure 1:**
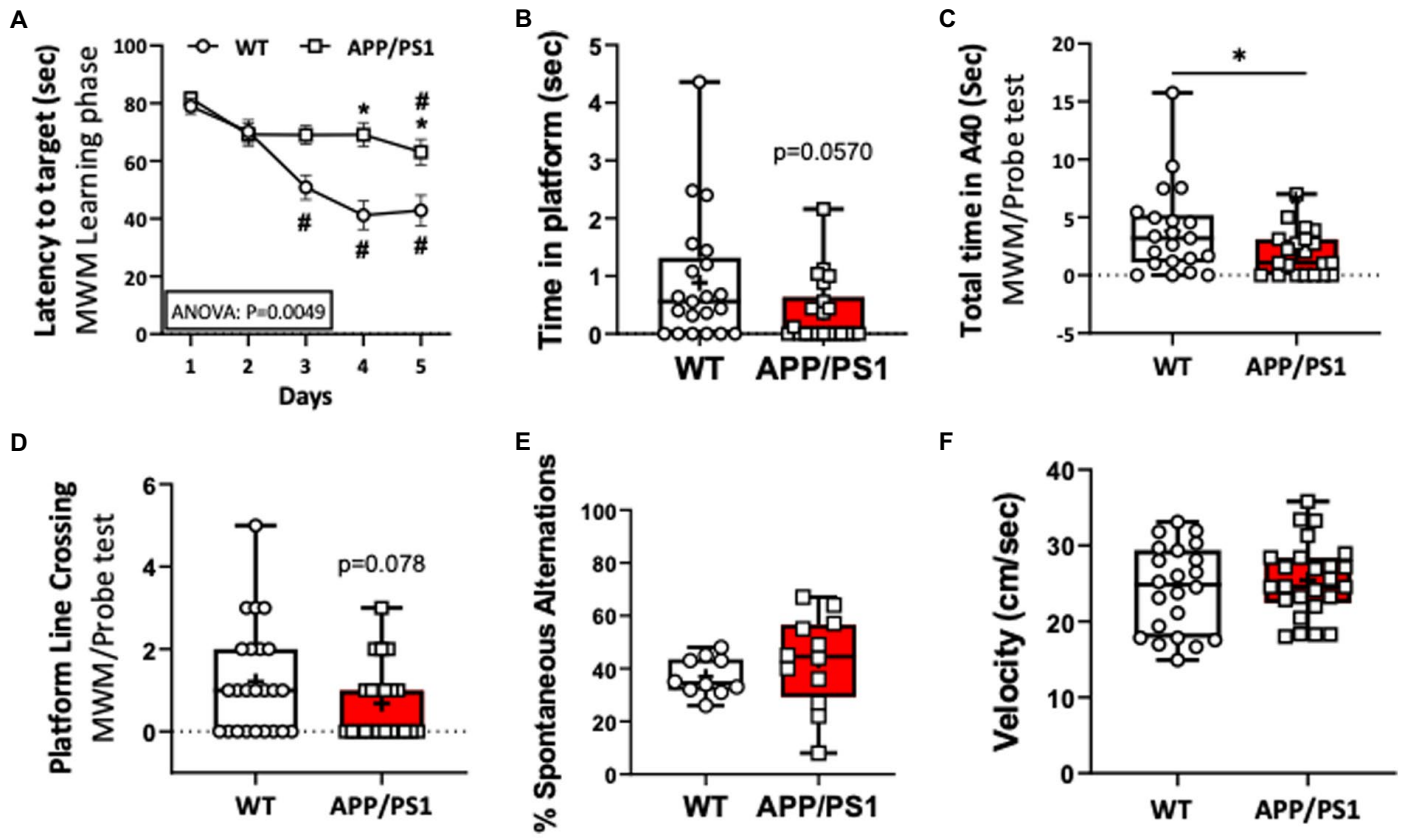
Impaired learning capacities of APP/PS1 mice in the Morris Water Maze test (MWM) compared to WT mice. **(A)** Increase in the latency (seconds) to reach the target reflected impaired learning capacities of APP/PS1 mice (*n*=22) compared to WT mice (*n*=24) in the 5 day training phase in MWM. *P < 0.05 vs WT; #*P* < 0.05 vs day 1. **(B,C)** APP/PS1 mice spent a reduced time in the platform **(B)** and in the target area **(C)**. **(D)** APP/PS1 mice had less platform line crossing compared to WT mice on the probe test. **(E)** The percent of spontaneous alternations in the Spontaneous Y-Maze test were not significantly different between groups. **(F)** There was no difference in velocity between WT and APP/PS1 mice during the testing. *P < 0.05 vs WT. Unpaired t test was used.

To confirm impaired short term spatial memory and learning function in the APP/PS1 AD mouse model, 9–10-month-old, female and male, APP/PS1 and WT mice were subjected to a Morris Water maze (MWM) test. We found that the individual latency curves of APP/PS1 and WT mice were similar on day 1 and 2 in the learning phase, indicating that the APP/PS1 mice did not differ in swimming ability, or with motivation to escape from the pool. From day 3 to 5, however, acquisition trials revealed a progressive and statistically significant decrease in latencies in APP/PS1 mice compared to WT mice (Figure 1A). During the probe trial, APP/PS1 mice spent longer times to reach the platform area, spent less time in the platform area upon finding it, and had a trend of less platform line crossings (Figures 1B–D). There were no differences between WT and APP/PS1 mice for the percentage of spontaneous alternations or in velocity (locomotion; Figures 1E,F).

### 3.2. APP/PS1 mice exhibit a reduced protein expression of ADAM17 in cerebral microvessels

**Figure 2:**
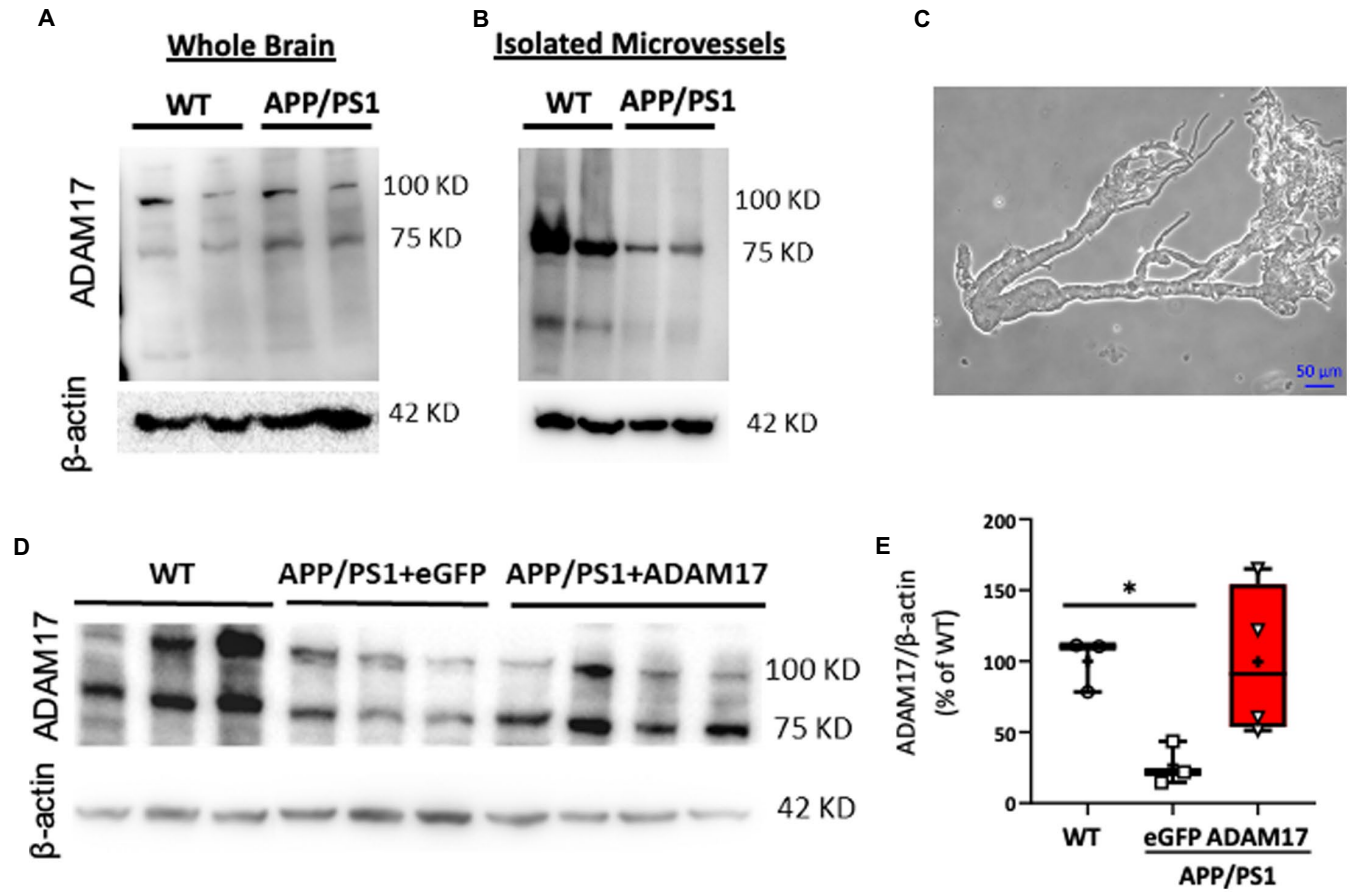
Reduced ADAM17 expression in cerebral microvessels of APP/PS1 mice is restored by ADAM17-AAV9. **(A)** ADAM17 expression was measured in whole brain lysates and **(B)** in isolated, purified cerebral microvascular preparations in WT and APP/PS1 mice. **(C)** Representative micrograph depicts a cerebral microvascular segment after centrifugation-based purification. **(D,E)** Representative image and quantification data of western immunoblots showing ADAM17 expression in cerebral microvessels of WT or APP/PS1 mice 3 months after injections with either eGFP-AAV9 or ADAM17-AAV9 virus. *n*=3−4. *P < 0.05. Two way ANOVA was used.

ADAM17 has been implicated in various cardiovascular pathologies, yet there have been only limited number of studies elucidating the role of vascular ADAM17 in neurogenerative diseases. Here, we found that while the protein expression of ADAM17 was similar in WT and APP/PS1 mouse whole brain lysates (Figure 2A), the expression of ADAM17 was remarkably reduced in purified cerebral microvessel preparations obtained from the APP/PS1 mice, when compared to those of WT mice (Figures 2B,C).

### 3.3. ADAM17 re-expression improves short-term memory and cognitive function in APP/PS1 mice

In order to delineate the role of vascular ADAM17 in impaired short-term memory in the APP/PS1 mice, we used a systemic, AAV9-mediated genetic delivery approach to increase ADAM17 expression. We found that 3 months after the delivery of the ADAM17-AAV9 construct, the protein expression of ADAM17 in cerebral microvessels of APP/PS1 mice was augmented compared to eGFP-AAV9 injected mice and that the expression level was similar to that measured in the cerebral microvessels of age-matched WT mice (Figures 2D,E).

**Figure 3:**
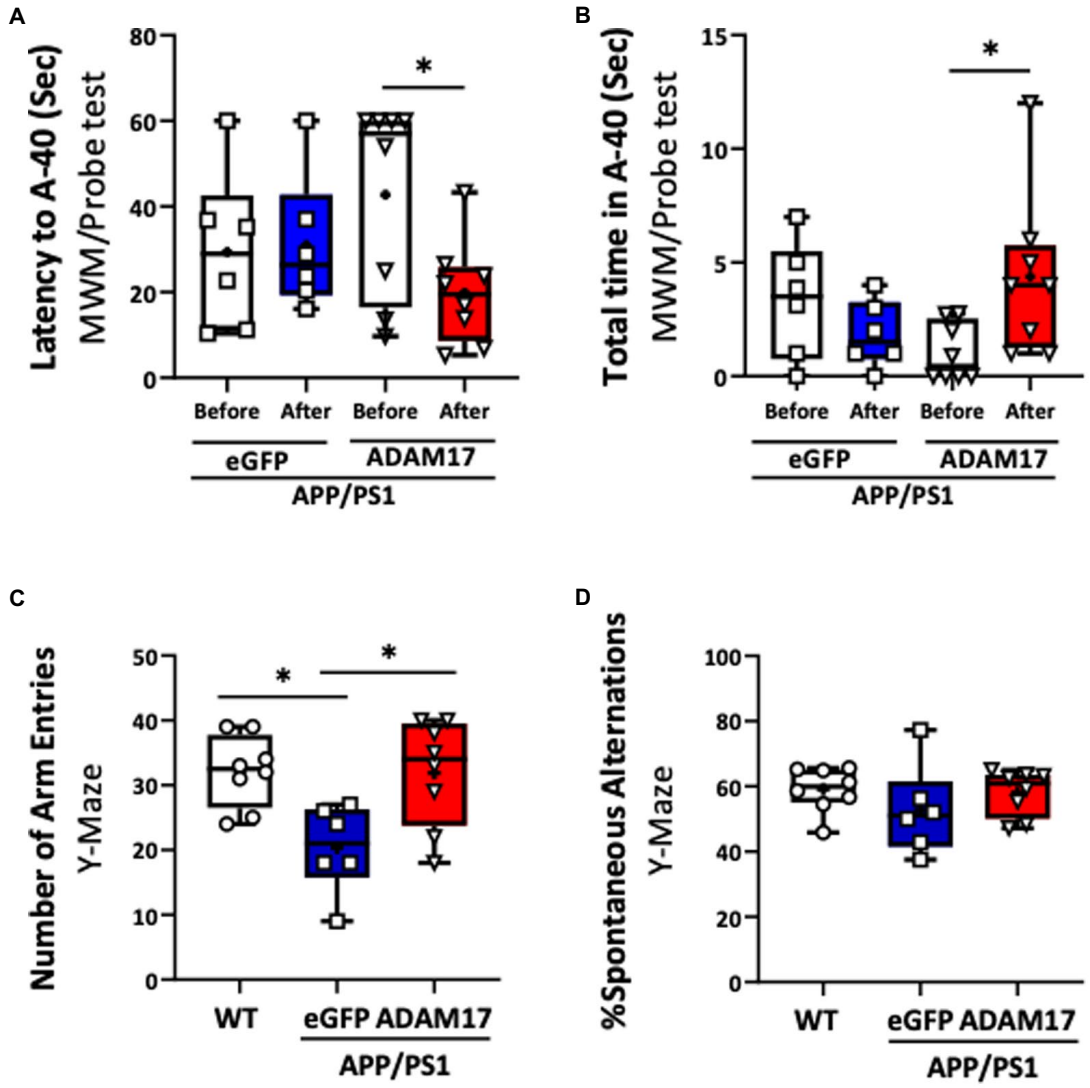
Increased ADAM17 expression improves cognitive function of APP/PS1 mice. **(A,B)** ADAM17 overexpressed APP/PS1 mice show a reduced latency (seconds) to reach the target area and increased total time in the target area compared to before ADAM17-AAV (adeno-associated virus) injection in a MWM probe test. There was no difference in APP/PS1 mice after receiving an eGFP-AAV9 injection. **(C,D)** The number of arm entries was lower in APP/PS1 mice with eGFP-AAV9 injected mice compared to the WT mice. This number was improved in the APP/PS1 mice with ADAM17-AAV9 virus injection. There was no difference in the percent of spontaneous alternation between the WT mice, eGFP, or ADAM17 overexpressed APP/PS1 mice in a spontaneous Y-Maze test. *P < 0.05. *n*=6−8. Unpaired t test was used.

We then determined the effect of ADAM17 re-expression on short-term memory and cognitive dysfunction in the APP/PS1 mice by performing multiple, independent behavioral tests. WT mice and APP/PS1 mice receiving eGFP-AAV9 served as controls. First, the ADAM17-AAV9 or eGFP-AAV9 injected mice were repeatedly subjected to the Morris Water Maze (MWM) test. We found that, 3 months after ADAM17 re-expression, APP/PS1 mice spent shorter times to reach the platform area (A-40) and spent longer times in the platform area, while APP/PS1 mice that received the eGFP-AAV9 injection displayed increased latency curves, similar to the level of APP/PS1 mice before the AAV injections (Figures 3A,B).

**Figure 4:**
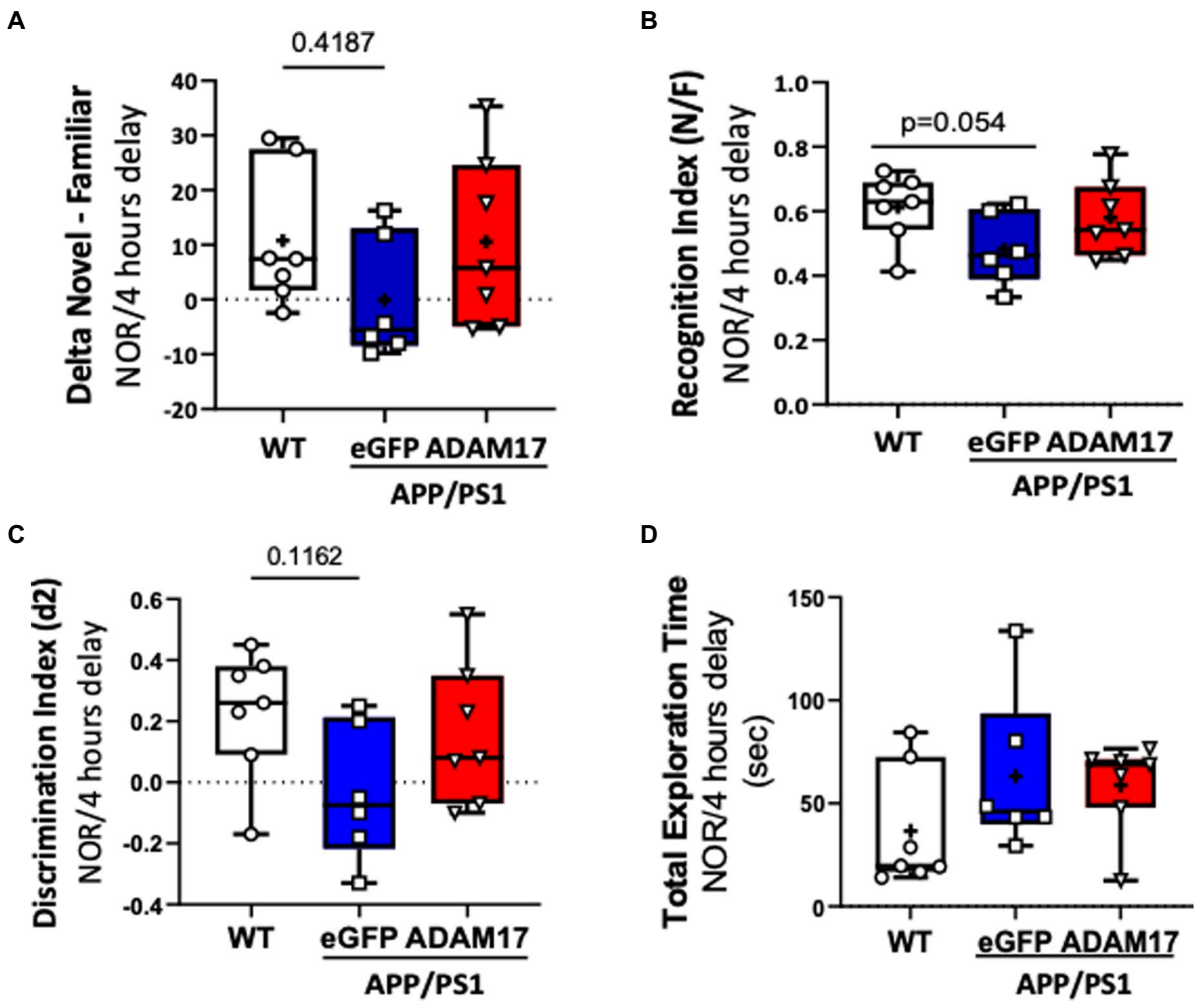
Increased ADAM17 expression in APP/PS1 mice improves performance in Novel Object Recognition test. **(A,B)** There was reduced time to explore the novel object and a reduced recognition index in the eGFP-AAV9 injected, but not in the ADAM17-AAV9 injected, APP/PS1 mice in a NOR test [Delta Novel – Familiar score, Recognition Index (Novel/Familiar, N/F)]. **(C)** There was decrease in the discrimination index (d2 ratio). **(D)** There was no change in Total Exploration Time between the three experimental groups. *n*=6−7. Two way ANOVA was used.

Mice in the experimental groups were additionally subjected to spontaneous Y-maze and Novel Objective Recognition (NOR) tests. In the spontaneous Y-maze, the eGFP-AAV9 injected APP/PS1 mice had a reduced number of arm-entries compared with the WT and ADAM17-AAV9 injected APP/PS1 mice (Figure 3C). There was no difference observed in the percent of spontaneous alternations between the three groups (Figure 3D). In the NOR test, compared to the age-matched WT mice, the APP/PS1 eGFP-AAV9 injected mice had a reduced delta novel-familiar score (Figure 4A), recognition index (novel/familiar, N/F) (Figure 4B) and discrimination index (d2 ratio) (Figure 4C). These parameters were similar in WT and the ADAM17-AAV9 injected APP/PS1 mice (Figures 4A–C). There was no significant difference in the total exploration time among the experimental groups (Figure 4D). Taken together, these observations indicates that re-expression of ADAM17 in APP/PS1 mice improved some of the indices of short-term memory and cognitive function to a level comparable to the aged-matched WT control mice.

### 3.4. Deposition of amyloid beta is not affected by ADAM17 re-expression in APP/PS1 mice

**Figure 5:**
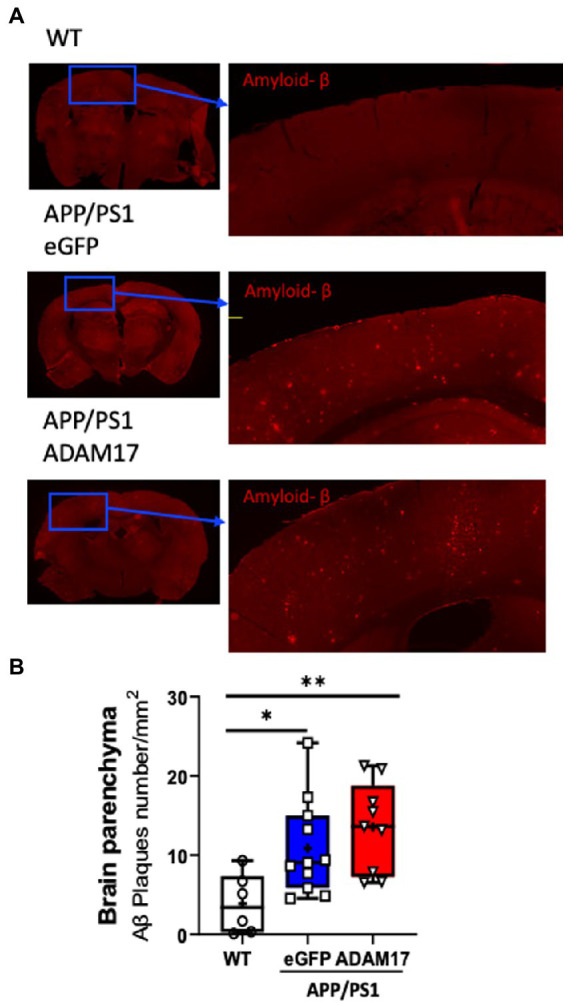
Amyloid-β plaque density was unchanged after ADAM17 re-expression in APP/PS1 mice. **(A,B)** Amyloid-beta (Aβ) plaques were identified *via* immunofluorescent staining and quantified. The number of Aβ plaques was significantly increased in the APP/PS1 mice and was unchanged with the ADAM17 injection. *n*=6−11. *p < 0.05 **p < 0.01. Two way ANOVA was used.

Brain sections of experimental mice were immunostained for amyloid beta (Aβ) for semi-quantitative assessment of cortical amyloidosis. When compared to age-matched WT mice, we found that there was a significant increase in the number of Aβ plaques in the APP/PS1 mouse cortex receiving the control eGFP-AAV9. Whereas we found no significant effect, i.e. decrease in the number of Aβ plaques, in the APP/PS1 mice injected with the ADAM17-AAV9 construct (Figures 5A,B).

### 3.5. ADAM17 re-expression improves cerebral artery vasodilator function in APP/PS1 mice

**Figure 6:**
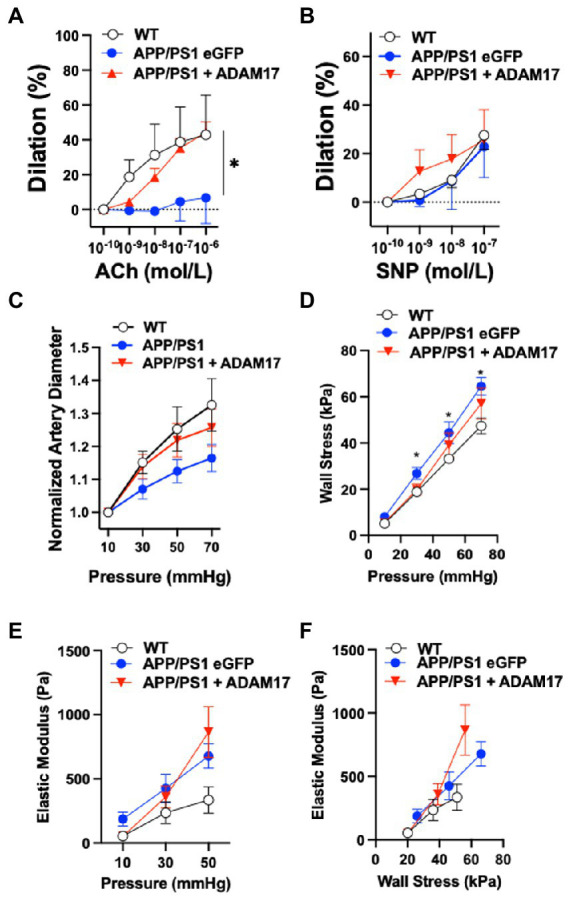
ADAM17 re-expression improves cerebral arteriole vasodilator function in APP/PS1 mice. **(A)** ADAM17 overexpressed APP/PS1 mice show an improved vasodilation in response to acetylcholine (ACh) in isolated basilar arteries compared to eGFP overexpressed APP/PS1 mice, which have a decreased dilatory response compared to the WT control mice. **(B)** There were no differences seen in the dilatory responses to sodium nitroprusside (SNP) in isolated basilar arteries of WT, eGFP, or ADAM17 overexpressed APP/PS1 mice. **(C)** Normalized (to 10 mmHg) diameter of arterioles in calcium free solution in response to incremental increases to intraluminal pressure (10 to 70 in 20 mmHg increments). **(D–F)** Calculated circumferential wall stress, incremental elastic modules and elastic modulus wall stress relationships in WT, eGFP-or ADAM17-overexpressed APP/PS1 mice. *n*=3−6 mice per group. *p < 0.05 WT, ADAM17 vs eGFP APP/PS1. Two way ANOVA was used.

We assessed cerebral artery vasodilator function and vascular biomechanics in WT, as well as eGFP-AAV9 and ADAM17-AAV9 injected APP/PS1 mice using isolated and pressurized cerebral arteries. We found that the endothelium-dependent vasodilation in response to acetylcholine (ACh) was significantly reduced in eGFP overexpressed APP/PS1 mice, when compared to WT controls (Figure 6A). ADAM17 re-expressed APP/PS1 mice showed an improved vasodilatory response to acetylcholine (ACh) compared to the eGFP overexpressed APP/PS1 mice; no significant difference was observed between the ADAM17 re-expressed APP/PS1 mice and WT mice (Figure 6A). There were no differences in the nitric oxide donor, sodium nitroprusside (SNP)-induced, vascular smooth muscle acting vasodilation in the isolated cerebral arteries of WT, eGFP, or ADAM17 overexpressed APP/PS1 mice (Figure 6B).

We also found that cerebral arteries of APP/PS1 (eGFP-AAV9) mice displayed a reduced passive diameter (in calcium free PSS and normalized to 10 mmHg) to incremental increases to intraluminal pressure (from 10 to 70 mmHg), when compared to WT controls (Figure 6C). These changes were accompanied by increases in artery wall circumferential stress and elastic modulus, as well as elastic modulus wall stress relationships in the APP/PS1 (eGFP-AAV9) mice (Figures 6D–F). Delivery and re-expression of ADAM17 in the APP/PS1 mice did not significantly affect these biomechanical properties of the cerebral arteries (Figures 6D–F). Taken together, these results indicate that re-expression of ADAM17 was associated with selective improvement of endothelial-dependent vasodilator function in the cerebral arteries of APP/PS1 mice.

### 3.6. ADAM17 re-expression in APP/PS1 mice had no effect on the vascularization of cerebral cortex

**Figure 7:**
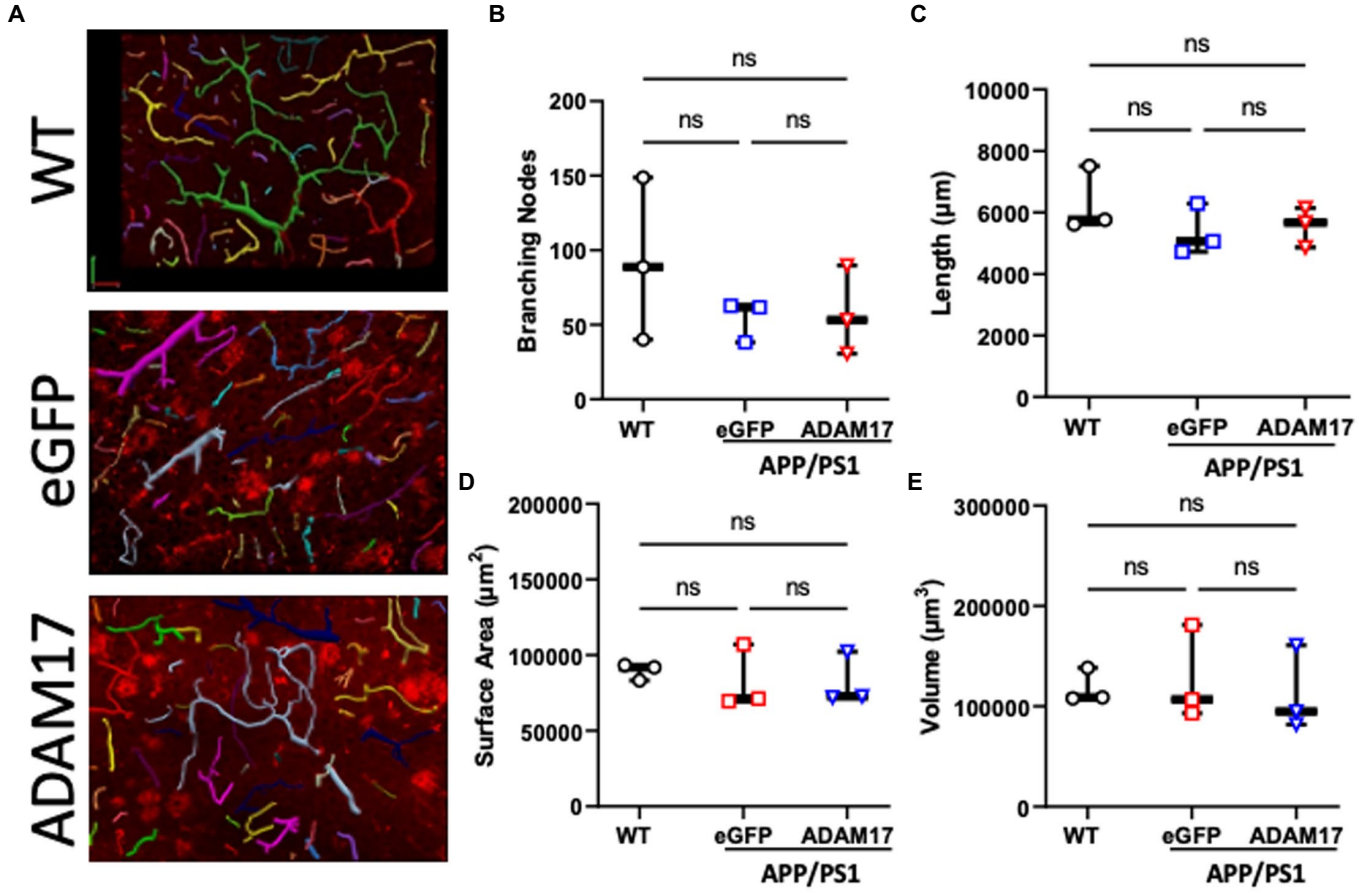
ADAM17 re-expression had no effect on the vascularization of the cerebral cortex in APP/PS1 mice. **(A)** Representative 3-dimensional reconstructions of the cerebral small vessel networks in WT, eGFP, and ADAM17 treated APP/PS1 mice. **(B–E)** No differences in the number of branching nodes, total vessel length, vessel surface area, or vessel volume was found between the three groups. Images were taken at 20X magnification, 0.5 μm slices in the z-plane. *n*=3 fields of view per mouse, 3 mice per group. *p < 0.05. Kruskal-Wallis test was used.

In order to quantify vascularization and microvessel density of the cerebral cortex, the vascular tree was immunofluorescently labelled and in thick sections (40 μm), z-stack images were used for 3-dimensional reconstruction followed by unbiased quantification of microvascular networks (Figure 7A). The microvascular networks in the WT, eGFP control, and ADAM17 re-expressed APP/PS1 groups showed no differences in the measured parameters, including, total vessel length, surface area, vessel volume, or the number of branching nodes (Figures 7B–E).

### 3.7. Proteomic profiling after ADAM17 re-expression in APP/PS1 mice

**Figure 8:**
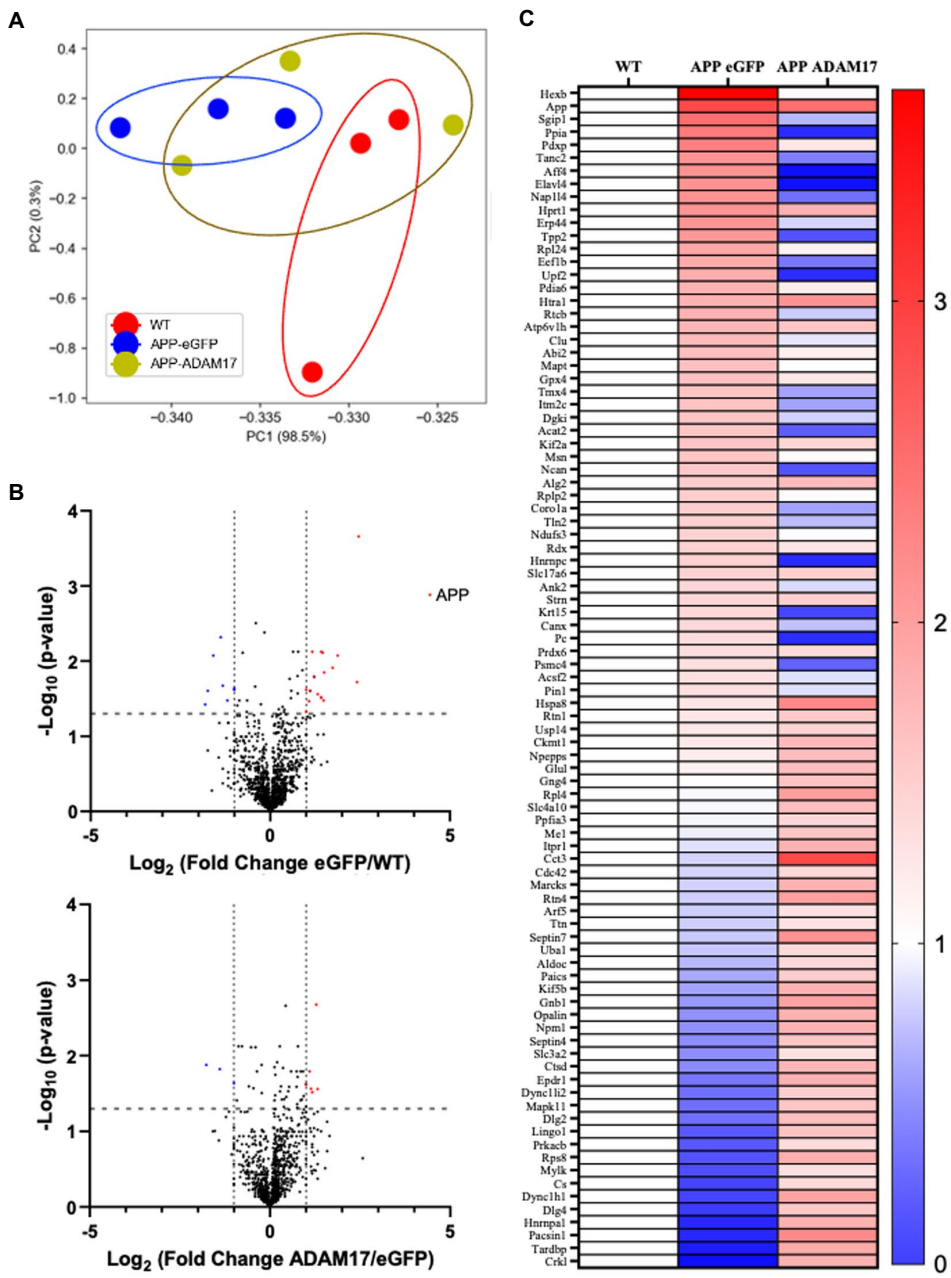
Proteomic profile of the brain of APP/PS1 mice. **(A)** A principal component analysis of proteins with ΣPSM per animal group ≥2, showing a difference in overall protein profiles between WT and eGFP mice, which was slightly corrected in the ADAM17 APP/PS1 mice. **(B)** Volcano plots of the protein profiles between WT/eGFP and eGFP/ADAM17 show the differentially up-and downregulated individual proteins. **(C)** Heatmap of the –log_10_(p-value) per group compared to WT show the up-and downregulated, significant proteins. 3 mice per group. *p < 0.05. Unpaired *t*-test was used.

We performed mass spectrometry analysis to identify proteins that were differentially expressed in the brain of the APP/PS1 mice, which were potentially affected by the AAV-mediated re-expression of ADAM17. Liquid chromatography mass spectroscopy (LC-MS) was performed in triplicate per group. First, we wanted to identify the general trend in the proteomic profile between WT, APP/PS1 + eGFP, and APP/PS1 + ADAM17. *Via* a principal component analysis (PCA), we saw a differential trend in the protein profile in APP/PS1 + eGFP mice versus the WT control mice. Whereby in APP/PS1 mice with ADAM17 re-expression, we saw an overall trend in the protein profile shifted towards the WT mice (Figure 8A). To further elucidate microvascular proteomic changes due to ADAM17 re-expression, we analyzed individual proteins identified *via* LC-MS, with a cut-off of a ΣPSM (peptide-spectrum match) of 2 per group (Figure 8B). Through these proteomic analyses, multiple proteins were identified as being significantly downregulated in the APP/PS1 + eGFP mice, that were augmented *via* ADAM17 re-expression, including numerous proteins that have been studied in relation to neurodegenerative diseases, such as Septin, Ankyrin-2 (Ank2), and Moesin (Msn), among others (Figure 8C).

**Figure 9:**
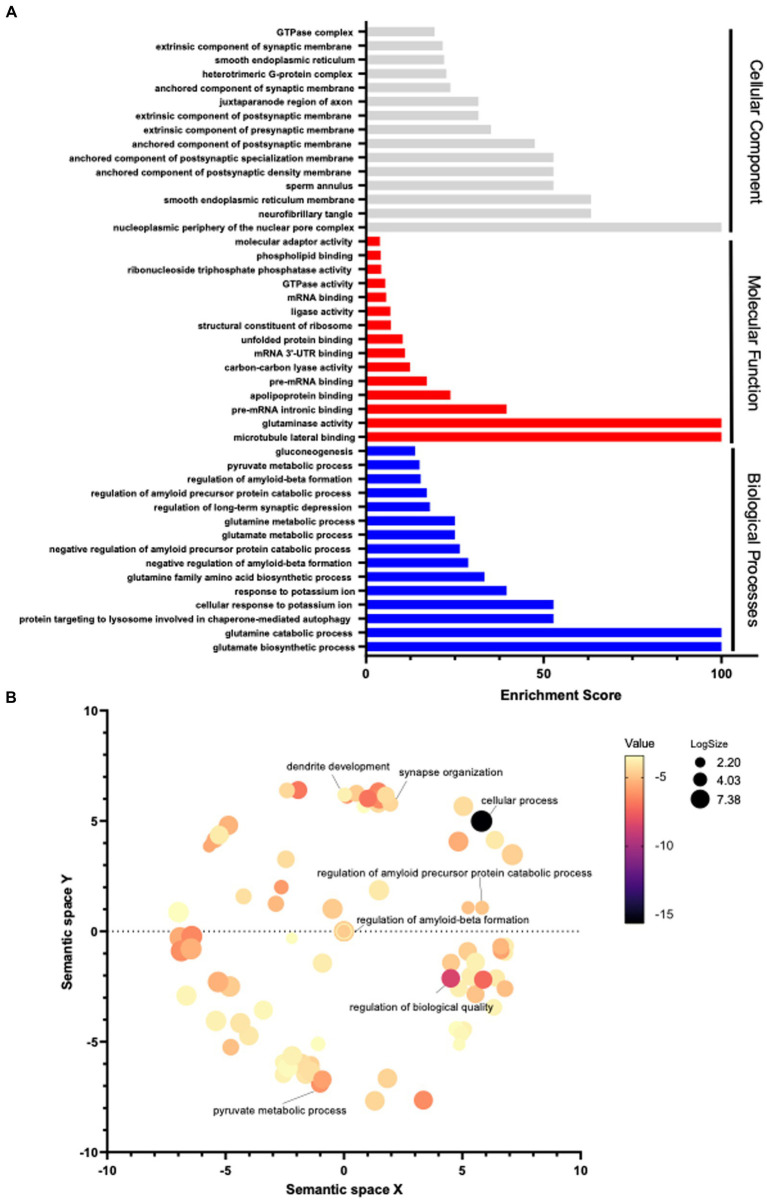
Pathway analysis in the brain of APP/PS1 mice. **(A)** Using the significantly differentiated proteins identified *via* proteomic analysis, a Gene Ontology pathway analysis was performed. Pathways with an FDR <0.05 were included, the top 15 significant pathways per category are graphed by enrichment scoring. **(B)** GO pathway analysis of biological processes was visualized by functional grouping. 3 mice per group.

Furthermore, Gene Ontology (GO) pathway analyses (Panther^®^) were completed in order to identify significant differential pathways that ADAM17 may impose its beneficial effects through. Using the significant identified proteins, biological processes, molecular functions, and cellular components were identified using an FDR of <0.05. The top 15 significant pathways per analysis are listed with the corresponding enrichment scores (Figure 9A). Moreover, these GO biological processes identified were mapped (Revigo^®^) to identify connected pathways, showing significant differences occur in pathways connected to amyloid precursor protein as expected, as well as in many metabolic pathways and in cellular organizational pathways (Figure 9B).

## 4. Discussion

The results from the present study indicate that cerebrovascular ADAM17 plays a role in the pathogenesis of Alzheimer’s disease (AD). This conclusion is supported by our results showing that short-term memory and cognitive dysfunction in the APP/PS1 mouse model of AD is associated with a reduced ADAM17 expression in cerebral microvessels, whereas re-expression of ADAM17 restored endothelium-dependent vasodilator function in cerebral arteries and improved memory and cognitive function in the APP/PS1 mice.

In this study, first we confirmed that 9-10 months old APP/PS1 mice (carrying transgenes for both APP bearing the Swedish mutation and PSEN1 containing an L166P mutation, both under the control of the Thy1 promoter) display short-term memory and cognitive deficits. Prior studies, including works from our laboratory on human autopsy findings, showed impaired microvascular vasodilator function in the presence of low and high AD neuropathological changes (Snowdon et al., 1997; Bagi et al., 2018; Park et al., 2018; Santisteban and Iadecola, 2018; Bagi et al., 2022). It is known that vascular impairments are highly prevalent in older adults with hypertension and type 2 diabetes (Prasad et al., 2017), which are risk factors for dementia and AD (Udelson, 2011; Goyal et al., 2016; Silverman et al., 2016). While clinical and preclinical studies argue that cerebrovascular endothelial dysfunction contributes to AD pathologies, the molecular mechanisms leading to cognitive decline remains largely unknown.

ADAM17 (also known as tumor necrosis factor (TNF)-α converting enzyme or TACE) exhibits α-secretase activity and has been implicated in APP cleavage, which results in a soluble, non-amyloidogenic fragment formation (Pietri et al., 2013). ADAM17 has multiple substrates, including mediators of vascular inflammation, inflammation resolution, and angiogenic factors (Gooz et al., 2009; Gooz, 2010). A previous study has found that a loss-of-function genetic variant in ADAM17 is associated with the pathogenesis of AD in humans (Hartl et al., 2020), whereas in rodent models genetic ADAM17 deletion caused an impaired collateral circulation formation and vascular growth in the cerebral surface arterioles (Lucitti et al., 2012). Thus, we hypothesized that in the microvasculature, ADAM17 could play an important role in the development of AD neuropathological changes and cognitive decline. Abnormal processing of APP is thought to promote AD development (Allinson et al., 2003; Sastre et al., 2008), among others, by causing cerebrovascular impairments (Sastre et al., 2008; Iadecola, 2017). In this study, we confirmed that β-amyloid plaque density is increased in the cerebral cortex of the APP/PS1 mice. However, we found that systemic delivery and AAV-mediated re-expression of ADAM17 in APP/PS1 mice improved cognitive functioning and endothelium-dependent vasodilator function (but not vascular wall biomechanics and cerebral vascular density), without affecting β-amyloid plaque density. These findings suggested beneficial effects of restoring microvascular ADAM17 expression in the APP/PS1 mice displaying already accumulated β-amyloid (Aβ) plaques. Based on our results we cannot confirm or exclude the possibility that a reduced microvascular ADAM17 expression *via* a reduced α-secretase activity and blunted non-amyloidogenic APP cleavage may have contributed to Aβ accumulation in the APP/PS1 mice, which has yet to be confirmed in longitudinal studies.

A common denominator in cerebrovascular disease and AD, and a likely contributor to microvascular dysfunction is chronic, low-grade inflammation (Borlaug and Paulus, 2011; Paulus and Tschope, 2013; Franssen et al., 2016). It is plausible that the role of ADAM17 can be also attributed to its ability to affect cerebrovascular inflammatory processes in AD mice. In this context, previous studies showed that deletion of TNFα reduces remyelination repair process in multiple sclerosis and cerebral ischemia mouse models, indicating a protective role for TNFα (Arnett et al., 2001; Lambertsen et al., 2009). ADAM17 not only sheds the membrane bound TNFα, but also cleaves both type I and type II TNFα receptors (TNFR_I_ and TNFR_II_). Interestingly, deletion of TNFR_I_ inhibits Aβ generation and prevents learning and memory deficits in AD mice (He et al., 2007; Lourenco et al., 2013). TNFR_II_, which has a higher affinity to the transmembrane TNFα and shows some anti-inflammatory effects, is considered to be protective in AD and ablation of TNFR_II_ impairs cognition in AD mice (Naude et al., 2014). On the other hand and in contrast to the aforementioned studies, deletion of both TNFR_I_ and TNFR_II_ accelerated AD (Montgomery et al., 2011), whereas TNFα inhibitors and an anti-TNF antibody (infliximab) improved cognitive decline in AD animal models and human patients (McAlpine et al., 2009; Shi et al., 2011). Collectively, these studies along with the results from our present findings suggest that a maintained ADAM17 activity, *via* regulating substrate availability, such as TNFα or TNFR_I_ and TNFR_II_ plays a role in AD pathogenesis. Future studies are needed to determine if reduced expression or re-expression of ADAM17 alters the proteolytic cleavage of TNFα or TNFR_I_ and TNFR_II_, whereby mediating the adverse or beneficial microvascular and cognitive effects in the APP/PS1 mice.

In addition, in this study we performed mass spectroscopy proteomic analysis in order to identify differentially expressed molecules involved in AD pathogenies, neurodegeneration, and repair; and test whether specific molecules and/or pathways were selectively reversed by ADAM17 re-expression. Through proteomic analysis, we identified multiple proteins that were significantly up or downregulated in the APP/PS1 mice, that were also changed, some back to normal levels, after ADAM17 re-expression. These proteins included Septin, Ankyrin-2, and Moesin, which have been previously implicated and studied in relation to neurodegenerative diseases, including AD. Furthermore, pathway analyses identified several candidate molecules that are associated with regulation of APP and β-amyloid formation and other biological and morphological quality regulatory proteins as well as neuronal morphogenesis and development. In this regard, further studies are needed to confirm whether these candidates can be associated or mechanistically involved in ADAM17-mediated regulation of microvascular and cognitive functioning in the APP/PS1 mice.

### 4.1. Limitations

There are several limitations to our study. It should be noted that our results provide no direct evidence for the mechanistic, causative link between impaired/improved vasodilation and reduced/restored ADAM17 expression in cerebral microvessels, which has yet to be elucidated and will be part of future studies. We did not measure overall, basal, or stimulated cerebral blood flow changes in experimental groups, which could also impact and determine abnormal cognitive functioning and the role of ADAM17 in the APP/PS1 mice. In addition, possible mechanisms independent from that of a microvascular mechanism in leading to impaired or improved cognitive functioning, which can also be related to ADAM17 in APP/PS1 mice, cannot be excluded. For example, we cannot exclude the role of astrocytes, microglia, or neuronal mechanisms, which we did not evaluate in this study. In this regard, it should be noted that we used systemic delivery and re-expression of ADAM17 in the APP/PS1 mice. Although we measured changes in ADAM17 protein expression in purified microvascular preparations, the expressional changes in other aforementioned cell types could have an impact on AD related neuropathological changes and memory and cognitive function in the APP/PS1 mice, which has yet to be delineated in future studies.

In conclusion, our study identifies a reduced expression of microvascular ADAM17 as a novel mechanism by which microvascular dysfunction occurs in an AD mouse model and by which it could contribute to the development of cognitive decline and memory deficits. We propose that targeting and potentially restoring normal activation of ADAM17 to improve microvascular endothelial function could be an effective approach, as it appears to improve some of the key molecular abnormalities previously implicated in AD pathogenesis.

## Data availability statement

We declare that the data supporting the findings of this study are available within the article and from the corresponding authors upon request. The mass spectrometry proteomics data have been deposited to the ProteomeXchange Consortium via the PRIDE partner repository with the dataset identifier PXD040026.

## Ethics statement

The animal study was reviewed and approved by IACUC at Augusta University.

## Author contributions

ZB conceptualized the project. ZB, YT, KF, HS, VB, LL, RW, and JF performed the experiments and analyzed data. YT, KF, RR, JF, and ZB wrote, edited, and approved the manuscript. JF and ZB supervised the research. All authors contributed to the article and approved the submitted version.

## Funding

This work was supported by awards from the National Institutes of Health, National Institute on Aging R01AG054651 to ZB and the T32HL155011 to KF.

## Conflict of interest

The authors declare that the research was conducted in the absence of any commercial or financial relationships that could be construed as a potential conflict of interest.

## Publisher’s note

All claims expressed in this article are solely those of the authors and do not necessarily represent those of their affiliated organizations, or those of the publisher, the editors and the reviewers. Any product that may be evaluated in this article, or claim that may be made by its manufacturer, is not guaranteed or endorsed by the publisher.
